# Cryptorchidism in Sarda Sheep: Incidence, Morphology, Ultrasonography and Behavioral Insights

**DOI:** 10.3390/ani16081253

**Published:** 2026-04-19

**Authors:** Charbel Nassif, Laura Mara, Fabrizio Chessa, Ignazio Cossu, Marilia Gallus, Federico Melis, Antonello Cannas, Maria Dattena

**Affiliations:** 1 Department of Agriculture, University of Sassari, 07100 Sassari, Italy; cannas@uniss.it; 2 Department of Animal Science, Agricultural Research Agency of Sardinia, 07100 Sassari, Italy; fchessa@agrisricerca.it (F.C.); icossu@agrisricerca.it (I.C.); mariliagallus@gmail.com (M.G.); federicomelis89@gmail.com (F.M.); mariadattena@gmail.com (M.D.)

**Keywords:** testicular development, ultrasonography, male infertility, sexual behavior, ram fertility, reproductive disorders, teaser rams, sheep reproduction

## Abstract

Cryptorchidism is the most common non-lethal congenital defect of the male reproductive system in sheep, with potential economic consequences for flock management. This study investigated the incidence, testicular morphology, ultrasonographic characteristics, semen quality, and sexual behavior of bilateral cryptorchid Sarda rams. Slaughterhouse inspections of 2360 lambs showed an incidence of 0.87% cryptorchidism. Cryptorchid testes were significantly rounder and lighter than intact testes, indicating impaired development in affected animals. Ultrasonography of 15 adult bilateral cryptorchid rams showed that retained testes were markedly undersized and that the left testis was less frequently visualized. No significant association with age was detected within the studied age range. All ejaculates recovered from bilateral cryptorchid rams were azoospermic. Nevertheless, behavioral trials suggested that bilateral cryptorchid males retained sexual interest and the ability to identify estrous ewes. These findings confirm the infertility of bilateral cryptorchid Sarda rams while highlighting their preserved sexual behavior, suggesting a potential zootechnical use as teaser rams for heat detection. Repurposing cryptorchid males in this way could represent a potential alternative to surgically modified teaser rams or the use of aprons on intact rams.

## Simple Summary

Cryptorchidism is a common reproductive disorder in male sheep in which one or both testes fail to descend into the scrotum. This condition can affect fertility and flock productivity. The present study investigated the frequency of cryptorchidism in Sarda sheep and evaluated the structure of the testes, ultrasound characteristics, semen quality, and sexual behavior of affected rams. Inspection of lambs at a slaughterhouse showed that cryptorchidism occurred in less than 1% of animals. The retained testes were smaller, rounder, and less developed compared with intact testes. Ultrasound examination confirmed that these testes remained small even in adult animals. Rams with both testes retained intra-abdominally did not produce sperm, confirming infertility. However, behavioral tests showed that these rams retained sexual interest and were able to identify estrous ewes under the conditions of this study. These findings suggest that although cryptorchid rams cannot be used for breeding, their potential use as “teaser” rams could be explored to help identify ewes ready for mating. Using bilateral cryptorchids in this way may represent a potential alternative to surgical procedures commonly used to produce “teaser males”.

## 1. Introduction

Cryptorchidism is the most common non-lethal genital defect in sheep [1,2]. Unilateral cryptorchid rams might be fertile, although their use for mating is not recommended, since this defect can be transmitted genetically. Thus, the general recommendation is their elimination from the flock [3,4]. In contrast, bilateral cryptorchidism occurs when both testes do not descend into the scrotal sac. While the scrotal sac provides a temperature lower than that of the rest of the body, allowing normal testicular development and function, retained testes that remain inside the body cannot produce spermatozoa, leaving the subject infertile [5,6].

Cryptorchidism is considered a multifactorial condition, arising from the interplay of genetic, hormonal, neural, and mechanical influences. Because the mechanisms regulating normal testicular descent are not fully understood, the causes of cryptorchidism remain only partly elucidated [7,8]. Current evidence suggests that factors such as prematurity, endocrine or genetic disorders, altered nerve signaling, and developmental mechanical issues can all contribute to the failure of proper testicular descent [9,10].

In sheep, the incidence of cryptorchidism differs between reports and breeds, with reported incidence ranging from 0.5% to 23.8% of males within affected flocks [6,7,11,12].

Testicular size in cryptorchid subjects in many species appears to be smaller than in healthy subjects [12,13,14,15]. Histological abnormalities of the testis have been reported in cryptorchid animals, including degeneration of seminiferous tubules and alterations in the number and size of Leydig cells, compared with intact animals in different species [6,14,15].

From a production standpoint, cryptorchid rams have no reproductive value and may negatively impact the income of farms producing breeding rams. These individuals are discarded and marketed at low prices for meat instead of generating higher value as breeding rams [12].

Despite the recognized reproductive and economic impact of cryptorchidism in sheep, its incidence and the associated anatomical, ultrasonographic, and behavioral characteristics in Sarda rams remain poorly documented. Moreover, no systematic studies have evaluated whether bilateral cryptorchid rams could provide any potential zootechnical advantage. In this context, this study also explored the possibility of repurposing naturally infertile bilateral cryptorchid rams as a safe and sustainable tool for detecting estrus in ewes. We hypothesized that cryptorchidism in Sarda sheep results in reduced testicular development and impaired spermatogenesis due to the abnormal intra-abdominal position of the testes, while sexual behavior may remain largely unaffected. Therefore, the aim of this study was to determine the incidence of cryptorchidism in Sarda lambs and to characterize the morphological, ultrasonographic, and reproductive features of retained testes, as well as to evaluate the sexual behavior of bilateral cryptorchid adult rams and their potential use as teaser males in flock management.

## 2. Materials and Methods

### 2.1. Study on Lambs from Slaughterhouse

#### 2.1.1. Incidence of Cryptorchidism

In Sardinia, the vast majority of lambs are slaughtered in their first month of life at around 10 kg of weight. Thus, to estimate the percentage of occurrence of cryptorchidism, visits to the slaughterhouse were organized during March and April. All procedures involving animals were conducted in accordance with national and institutional guidelines for the care and use of animals in research. The study protocol was approved by the Organismo Preposto al Benessere e alla Sperimentazione Animale (OPBSA) (protocol no. 53034, 22 May 2025). A total of 2360 lambs of both sexes were examined. After slaughter, the suspended lamb carcasses were examined for sex to determine the ratio of females to males, then males were examined by palpation of the scrotum to determine the presence or absence of testes in the scrotal sac. When a cryptorchid lamb was found it was marked and the testes collected after evisceration. For each cryptorchid lamb found, testes from an intact lamb randomly selected were also collected to serve as controls. Incidence of cryptorchidism was calculated as a proportion, with 95% confidence intervals estimated using the exact binomial method.

#### 2.1.2. Lamb Testis Measurements

The collected testes were transported to the laboratory in a cooled container (4 °C) and subsequently weighed and measured. Out of 22 lambs, 20 had both testes measured. Testes from one bilateral cryptorchid and one intact lamb were eliminated because they were incorrectly harvested and were therefore not measurable. One testis presenting pathological alterations was excluded from morphometric analyses. Testicular dimensions were measured using precision vernier calipers: D1 (longitudinal length), D2 (longitudinal length including epididymis), and D3 (transverse width). The operator performing measurements and exclusions was blinded to the group (intact vs. cryptorchid) to minimize bias. Each testis (including epididymis) was also weighed at collection. From these measurements, three shape descriptors were derived. Aspect ratio was calculated as width divided by length, with values approaching 1 indicating rounder shapes. Eccentricity was calculated from the semi-major and semi-minor axes of an ellipse, where values closer to 0 indicate circularity. Circularity was defined as 4π*A*/*P^2^*, with area (*A*) estimated as π*ab* and perimeter (*P*) approximated using Ramanujan’s formula:

$$P\approx \pi [3(a+b)-\sqrt{\left(3a+b)(a+3b\right)}]$$

where *a* and *b* are the semi-major and semi-minor axes, respectively. This approach represents a two-dimensional geometric approximation of testicular shape based on linear caliper measurements.

Because each animal contributed two testes, statistical analyses were performed at the testis level using linear mixed-effects models, with group (Intact vs. Cryptorchid) included as a fixed effect and animal identity as a random intercept. Separate models were fitted for each outcome variable. Estimated marginal means were obtained from the fitted models, and group comparisons were performed accordingly. Effect sizes were calculated as Hedges’ g with 95% confidence intervals. Statistical significance was set at *p* < 0.05. Model assumptions were assessed by visual inspection of residuals and Q–Q plots.

### 2.2. Study of Adult Bilateral Cryptorchid Rams

To further investigate the morphological and behavioral characteristics of bilateral cryptorchid rams, 15 adult animals aged between 1.5 and 6 years from the Agris Sardegna research center in Bonassai (Sassari, Sardinia, Italy; 40°40′22.74″ N, 8°21′50.86″ E) were included in the study and maintained under semi-intensive experimental conditions. All procedures involving animals were conducted in accordance with national and institutional guidelines for the care and use of animals in research. The study protocol was approved by the Organismo Preposto al Benessere e alla Sperimentazione Animale (OPBSA) (protocol no. 53034, 22 May 2025).

#### 2.2.1. Adult Testis Measurements

Animals were divided into two groups: a control group consisting of intact rams (*n* = 7) and a treatment group consisting of bilateral cryptorchid rams (*n* = 15), both within a similar age range (1.5–6 years).

In the control group, scrotal testes were measured using Vernier calipers. Ultrasonography was used to identify the testis–epididymis junction corresponding to the D2 measurement. Three linear dimensions (mm) were recorded: D1 (longitudinal length), D2 (longitudinal length including the epididymis), and D3 (transverse width).

The same definitions were applied to both intact and bilateral cryptorchid rams. However, measurements were obtained using different modalities (calipers in intact rams and ultrasonography in bilateral cryptorchid rams); therefore, comparisons between groups should be interpreted with caution.

In the treatment group, ultrasonography was performed using an Esaote MyLab™ Omega system (Esaote SpA, Genoa, Italy; operating system based on Windows^®^ 10) with an abdominal convex probe (Esaote AC2541). Animals were restrained using a sheep tilt table without sedation. Standard abdominal scanning settings were applied, with imaging parameters (including gain and depth) adjusted as needed to optimize visualization of the testes and surrounding structures. The probe was applied with gentle pressure in the abdominal and inguinal regions to locate the retained testes, and three linear dimensions (D1–D3, mm) were recorded. Testes not visualized were coded as “not found” and excluded from size analyses but included in detection-rate calculations. All examinations were performed by a single experienced operator using the same equipment throughout the study. Operator blinding was not feasible due to the evident clinical condition of the animals, and inter-observer repeatability was not assessed. Age was calculated from date of birth to the day of ultrasonographic examination.

Adult testicular measurements were analyzed using linear mixed-effects models with animal identity included as a random intercept to account for repeated measurements from the same ram. For bilateral cryptorchid rams, separate models were fitted for D1, D2, and D3 including side and age as fixed effects. For group comparisons, models included group and side as fixed effects. Estimated marginal means were obtained using the emmeans package. Effect sizes for group comparisons were calculated as Hedges’ g with 95% confidence intervals. Model assumptions were assessed by visual inspection of residuals and Q–Q plots.

In both groups, aspect ratio, eccentricity, and circularity were calculated and analyzed as previously described for lambs. Assumptions of normality and homogeneity of variance were assessed using Shapiro–Wilk and Levene’s tests, respectively.

Testicular weight was not recorded in adult rams because measurements were obtained in vivo using ultrasonography, which does not allow direct determination of testicular mass.

#### 2.2.2. Seminal Collection

To confirm azoospermia, seminal fluid was collected from the 15 bilateral cryptorchid adult rams maintained at the Agris Sardegna research farm. Twenty females from the same farm were hormonally synchronized using intravaginal progesterone-impregnated sponges (Syncro-Part^®^, Ceva Santé Animale, Libourne, France) for 14 days, followed by the administration of 500 IU eCG (Syncro-Part^®^, Ceva Santé Animale, Libourne, France) at sponge removal. Estrus was assumed to occur 36 h after sponge withdrawal. The females were then divided into four groups of five ewes each. A single bilateral cryptorchid ram was introduced to each group and allowed to mount and service one female. Immediately after service, the female was restrained and the ejaculate was recovered from the vaginal cavity using a speculum. For each ram, a single ejaculate was collected. The recovered volume was variable, ranging from 0.6 to 1.1 mL, reflecting the nature of the collection method. The ejaculate was immediately transported to the laboratory and examined using a Computer Assisted Sperm Analysis (CASA) system (Ceros II, Hamilton-Thorne, Beverly, MA, USA; software version 1.13.7). Briefly, a 10 µL aliquot was evaluated on a pre-warmed (37 °C) slide (Leja slides, 20 µm; IMV Technologies, L’Aigle, France). CASA was performed using standard settings for ovine semen, including a frame rate of 60 Hz and 30 frames per acquisition. The analysis was specifically focused on detecting the presence or absence of spermatozoa rather than performing a full quantitative or motility assessment; therefore, no minimum concentration threshold was defined. Accordingly, the evaluation was designed as a qualitative assessment aimed at confirming the absence of spermatozoa, rather than a comprehensive semen analysis. The entire slide area was systematically scanned across multiple microscopic fields, and evaluation was considered complete only after full examination of the sample to confirm the absence of spermatozoa.

#### 2.2.3. Behavior Towards Females

To evaluate the sexual behavior of bilateral cryptorchid Sarda rams and their ability to detect females in estrus, ten bilateral cryptorchid rams aged 2–3 years from the Agris Sardegna experimental farm were used. Rams were tested individually to avoid male–male competition. Trials were conducted on the same day under similar management, housing, and feeding conditions (300 g concentrate and hay ad libitum). The order of testing was based on animal availability and was not randomized. All animals showed normal temperament, and environmental conditions were comparable across sessions. The number of animals used for behavioral assessment was limited by the availability of adult bilateral cryptorchid rams within the research facility, and the study was therefore conducted as an exploratory evaluation. Each ram was introduced into a test pen (10 × 4 m) containing a group of five adult ewes: one in estrus induced by hormonal synchronization using the protocol described in the previous section (progesterone sponges + eCG) and four ewes not in estrus serving as controls. Behavioral observations began immediately after the introduction of the ram and were recorded for a maximum duration of 5 min. The following behavioral parameters were recorded: approach toward the estrous ewe and flehmen response, as well as reaction time to mount, defined as the time elapsed from the introduction of the ram into the pen to the first mounting attempt toward the ewe in estrus, and reaction time to service, defined as the time from the introduction of the ram into the pen to successful copulation. Once the ram serviced the ewe, the observation period was terminated and the ram was removed from the pen.

## 3. Results

### 3.1. Study on Lambs from Slaughterhouse

#### 3.1.1. Incidence of Cryptorchidism

**Figure 1:**
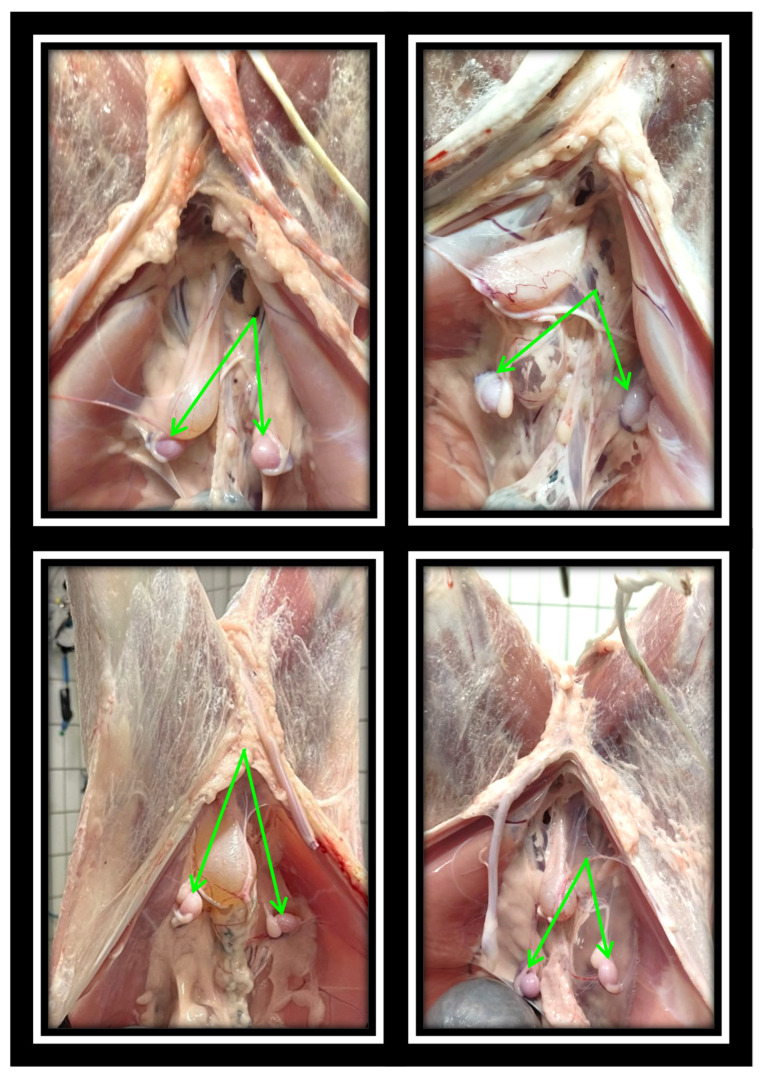
Post-evisceration images of lambs at the slaughterhouse showing bilateral cryptorchidism, with intra-abdominal testes indicated by green arrows.

**Figure 2:**
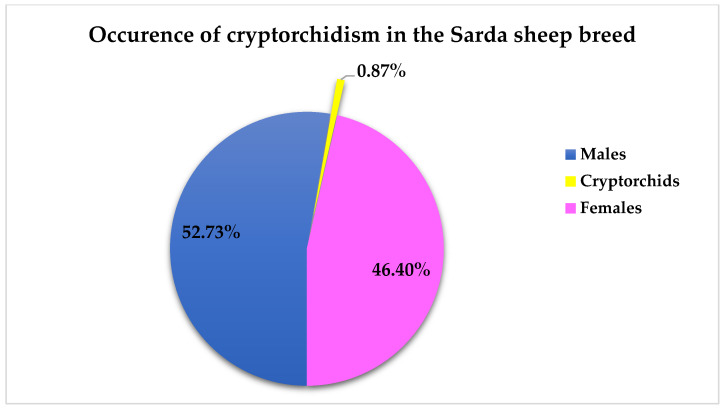
Distribution of lambs by sex and incidence of cryptorchidism in a slaughterhouse population of Sarda sheep.

Out of 2360 Sarda lambs inspected on 4 different slaughterhouse visits, 1095 (46.4%) were females and 1265 (53.6%) were males. Within the male population, we found 11 bilateral cryptorchid lambs (Figure 1), corresponding to 0.87% (95% CI: 0.43–1.55%) of males being cryptorchids (Figure 2). In our sample no unilateral cryptorchid lambs were found.

#### 3.1.2. Lamb Testis Measurement

**Figure 3:**
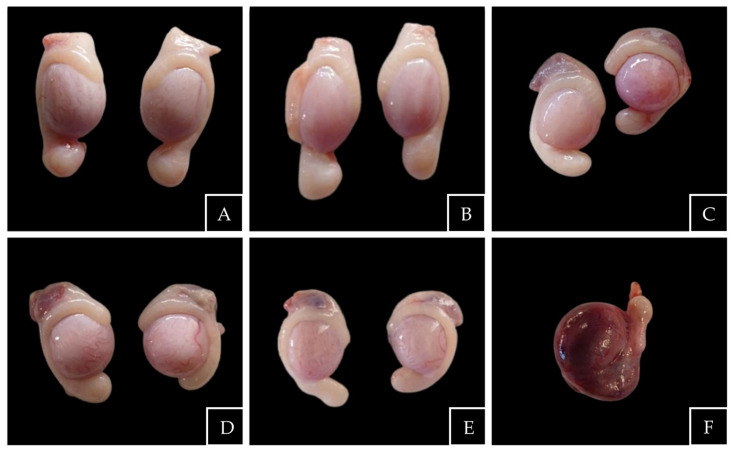
Testicular morphology in lambs. (**A**,**B**) Testes from intact lambs showing normal elliptical shape; (**C**–**E**) testes from cryptorchid lambs displaying a more rounded morphology; (**F**) hemorrhagic testis observed in a cryptorchid lamb.

Testicular morphometry in lambs revealed significant differences between intact and bilaterally cryptorchid animals for selected dimensions and shape descriptors (Table 1, Figure 3).

Among linear dimensions, testicular length including the epididymis (D2) was significantly lower in bilateral cryptorchid lambs compared with intact controls (22.17 ± 2.15 vs. 24.65 ± 2.06 mm; *p* = 0.015). No significant differences were observed for D1 (18.89 ± 2.73 vs. 20.10 ± 1.86 mm; *p* = 0.22) or D3 (13.42 ± 1.87 vs. 13.20 ± 1.51 mm; *p* = 0.73). Testicular weight was significantly lower in cryptorchid lambs compared with intact controls (2.90 ± 0.94 vs. 3.67 ± 0.71 g; *p* = 0.037).

Shape descriptors differed significantly between groups. Cryptorchid testes showed a higher aspect ratio (0.71 ± 0.06 vs. 0.66 ± 0.05; *p* = 0.010; Hedges’ g = −1.04), higher circularity (0.96 ± 0.02 vs. 0.93 ± 0.02; *p* = 0.017; Hedges’ g = −0.99), and lower eccentricity (0.70 ± 0.06 vs. 0.75 ± 0.04; *p* = 0.008; Hedges’ g = 1.05) compared with intact testes.

### 3.2. Study of Adult Bilateral Cryptorchid Rams

#### 3.2.1. Adult Testis Measurements

**Figure 4:**
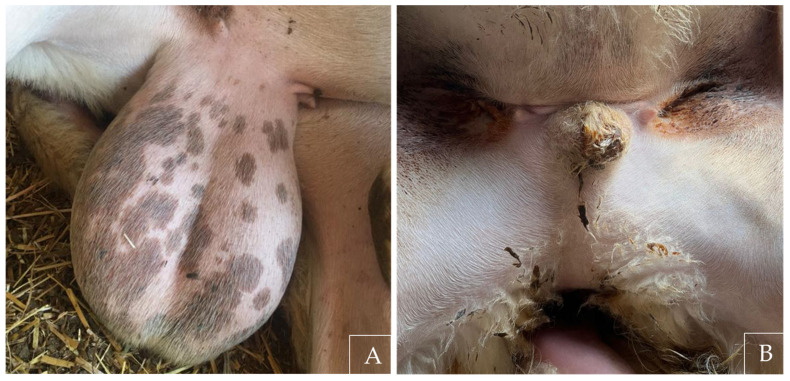
External appearance of reproductive organs in (**A**) intact ram, showing testes present in the scrotal sac, and (**B**) cryptorchid ram, showing an empty scrotal sac.

**Figure 5:**
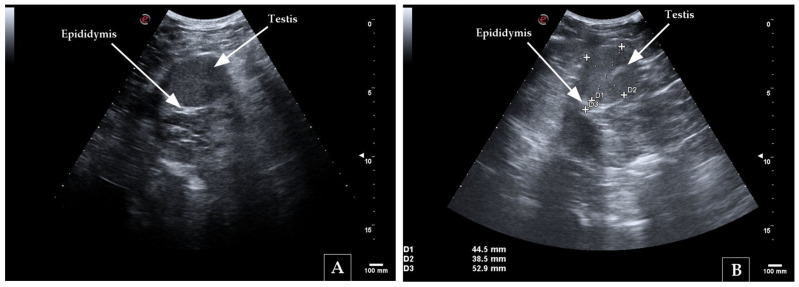
Ultrasonographic images of intra-abdominal testes in two bilateral cryptorchid rams. (**A**) Retained testis visualized within the abdominal cavity; (**B**) Testis with linear measurements (D1–D3) and identification of the epididymis. The scale on the right indicates 100 mm.

Ultrasonography was performed on all 15 bilaterally cryptorchid rams (Figure 4). Ultrasonographic examination (Figure 5) allowed the identification and localization of intra-abdominal testes and in vivo measurement of their dimensions. The right intra-abdominal testis was identified in all animals, whereas the left testis was not visualized in 4/15 cryptorchid rams (26.7%). Linear mixed-effects models showed no significant effect of side on testicular dimensions in bilateral cryptorchid rams (all *p* > 0.10). Estimated marginal means indicated similar values between left and right testes for all measurements (D1, D2, and D3). Similarly, no significant effect of age on testicular dimensions was detected (all *p* > 0.10) within the studied range (1.5–5.5 years).

When compared with intact rams, mixed-effects models showed that testicular dimensions were significantly lower in bilateral cryptorchid rams (all *p* < 0.001; Table 2). Estimated marginal means were markedly lower in cryptorchid than in intact rams, with values of 45.45 vs. 103.50 mm for D1, 54.11 vs. 160.93 mm for D2, and 30.18 vs. 73.00 mm for D3, corresponding to mean differences of −57.60 mm (95% CI: −65.06 to −50.12 mm), −106.03 mm (95% CI: −115.20 to −96.93 mm), and −42.68 mm (95% CI: −46.57 to −38.78 mm), respectively. Effect sizes were extremely large (Hedges’ g = 6.70–9.41).

In contrast, no significant differences were observed between groups for aspect ratio, eccentricity, or circularity (all *p* > 0.05; Table 3), with small effect sizes (Hedges’ g ≤ 0.46).

#### 3.2.2. Seminal Analysis

**Figure 6:**
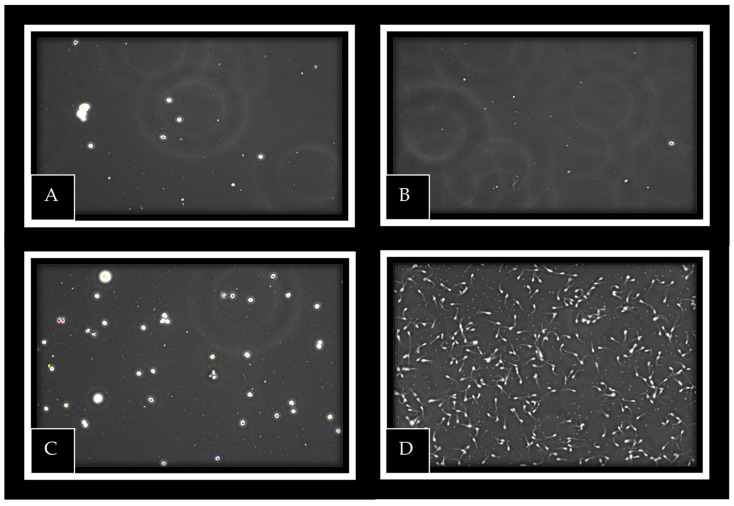
Microscopic evaluation of semen samples. (**A**–**C**) Ejaculates from bilateral cryptorchid rams showing absence of spermatozoa; (**D**) ejaculate from an intact ram showing normal sperm presence.

Out of the 15 bilateral cryptorchid rams, 14 completed a full mount and service on the synchronized females. The ejaculate deposited in the vagina was recovered for all 14 males and assessed microscopically. No spermatozoa were detected in any recovered ejaculate (Figure 6).

#### 3.2.3. Behavior Towards Females

**Figure 7:**
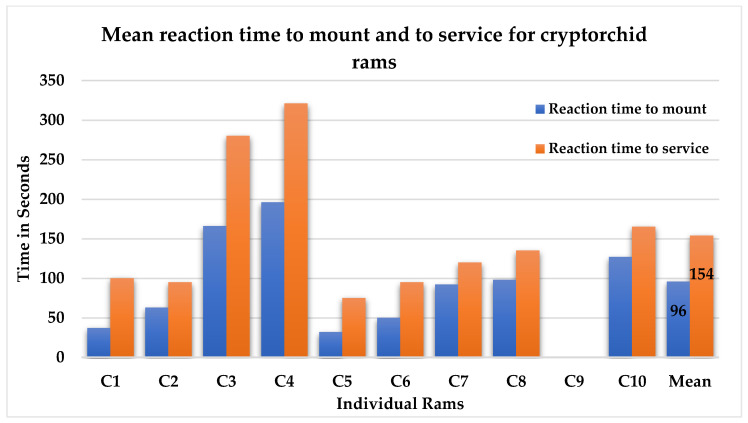
Reaction time to mount and to service in individual bilateral cryptorchid rams and corresponding mean values (C = bilateral cryptorchid males).

Nine of ten bilateral cryptorchid rams actively approached females, displayed a flehmen response, and were able to detect and complete a successful mount on estrous females. Only 20% of males hesitated at first before attempting to mount the females. Only one ram did not show sexual interest during the observation period and remained distracted by the handler. The reaction time to mount was 96 ± 57.7 s (range: 32–196 s), while the reaction time to service was 154 ± 87.7 s (range: 75–321 s) (Figure 7).

## 4. Discussion

The incidence of cryptorchidism in the Sarda breed, as observed at the slaughterhouse, was relatively low (0.87%). This value is consistent with reports in some sheep populations, where incidence ranges from 0.5 to 1% [4,6,7], whereas higher rates (up to 10–23%) have been reported in other breeds and production systems [11,12]. Such variability may reflect differences in genetic background, breed susceptibility, and environmental or management factors across geographical regions.

The incidence reported in the present study is based on lambs sampled during the March–April slaughter period, which predominantly includes offspring from primiparous ewes. This sampling strategy may introduce seasonal and parity-related biases and does not ensure a strictly random population, potentially limiting representativeness at the breed level. These limitations should be considered when interpreting the results. Future studies should assess seasonal variation in cryptorchidism incidence, particularly in winter, when lambs from multiparous ewes are more prevalent.

This study demonstrated that testes from cryptorchid lambs are both rounder and lighter than those of intact lambs, despite showing no substantial differences in simple linear dimensions. The three independent shape descriptors (aspect ratio, eccentricity, and circularity) indicated a more spherical morphology. This pattern is consistent with impaired longitudinal growth, with retained testes adopting a more spherical shape and reduced mass, as previously reported [6,7,16].

**Figure 8:**
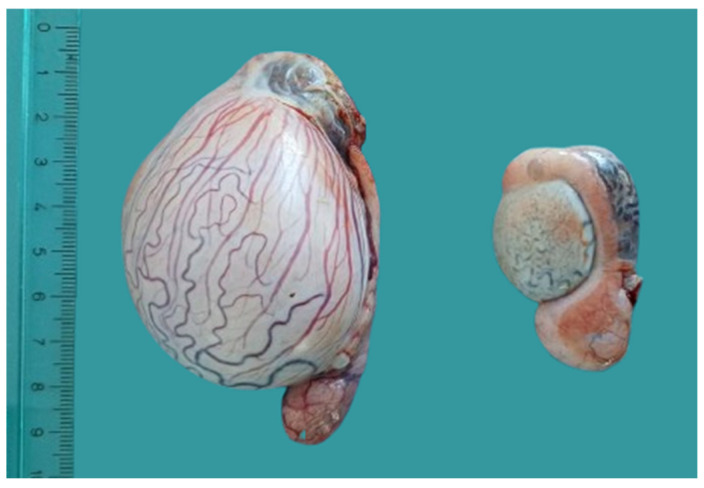
Testes collected from a bilateral cryptorchid ram, showing marked size asymmetry and epididymal malformation.

In adult rams, ultrasonography showed that testicular size did not differ significantly between sides when both testes were detected. Testes remained undersized, with no detectable association between age and size within the studied range [17,18]. The left testis was more frequently not visualized, consistent with observations reported in other species, including humans, where atrophic testes may escape ultrasonographic detection [19]. In our study, one cryptorchid ram died prior to ultrasonographic examination, allowing direct post-mortem evaluation of the testes (Figure 8). One testis corresponded to ultrasonographic measurements while the contralateral testis was severely atrophic and both exhibited epididymal malformations. This observation is consistent with the known limitations of ultrasonographic detection [20].

Overall, testes of bilateral cryptorchid rams were markedly smaller than those of intact rams (*p* < 0.0001; Hedges’ g = 6.59–9.99), consistent with heat-induced impairment of spermatogenesis [5,6,21]. In contrast, shape descriptors did not differ significantly between groups. This may indicate attenuation of shape differences with age; however, this finding may also reflect methodological differences between post-mortem measurements in lambs and ultrasonographic measurements in adult rams. This finding is consistent with previous reports indicating that, although cryptorchid testes are markedly reduced in size, their overall shape may remain comparable to that of intact testes in adult animals, reflecting a uniform atrophic process rather than differential morphological remodeling [7,14]. In this context, testicular degeneration associated with prolonged intra-abdominal retention primarily affects volume and mass rather than gross geometric proportions. Therefore, caution is required when interpreting apparent age-related changes in testicular morphology across these datasets, as morphological differences appear more evident during early development, while comparisons between datasets remain influenced by methodological differences.

This discrepancy likely reflects both biological and methodological factors, as post-mortem evaluation allows precise assessment of early hypoplasia, whereas ultrasonography may underestimate size and does not provide testicular weight. Chronic exposure to abdominal temperature may also stabilize testicular morphology over time. All adult bilateral cryptorchid rams originated from a single research center, which may limit generalizability. Further studies are needed to confirm these findings in broader populations.

As for semen collection, all recovered samples from bilateral cryptorchid rams contained no detectable spermatozoa, consistent with azoospermia. Even mild, sustained warming of the mammalian testes arrests spermatogenesis via germ-cell apoptosis and tubular degeneration, leading to failure of sperm production and azoospermia at ejaculation [18,22]. Unilateral cryptorchid rams may remain fertile due to the descended testis, although they are typically excluded from breeding [23]. Semen collection via vaginal recovery is not standardized and may introduce variability; therefore, future studies should include more robust methods such as testicular biopsy or epididymal sperm aspiration.

Despite the absence of detectable spermatozoa, bilateral cryptorchid rams displayed preserved sexual behavior. In the present study, most rams actively identified and mounted estrous ewes, suggesting the ability to discriminate females in heat. Reaction time to service was shorter than values previously reported for intact rams (154 vs. 330 s [24]), and similar animals have been shown to induce a ram effect in ewes [25] A possible explanation is that Leydig cell steroidogenesis is less sensitive to elevated temperature than spermatogenesis, allowing maintenance of androgen production and sexual behavior despite testicular dysfunction [16,26]. In sheep husbandry, sterile teaser males are sometimes produced using methods such as the short-scrotum technique, which suppress spermatogenesis through increased temperature while preserving androgen-dependent behavior [16,27]. However, these observations should be interpreted cautiously, as the behavioral assessment was exploratory and conducted on a limited number of animals. Further investigation, particularly of endocrine parameters, is needed to better explain the preserved sexual behavior observed in cryptorchid rams.

From a management perspective, cryptorchidism can be identified by scrotal palpation, even from early age, with ultrasonography providing confirmation of retained testes. Early identification may allow culling or selective retention of bilateral cryptorchid rams for potential use as teaser rams. Unilateral cryptorchid rams should always be excluded from breeding programs.

In the context of estrus detection, bilateral cryptorchid rams may represent a potential alternative to surgically modified teaser rams. However, their suitability should be evaluated individually, particularly regarding sexual behavior and detection efficiency. The optimal ram-to-ewe ratio remains unknown and may vary with management conditions. Practical limitations, including health risks and behavioral variability, should also be considered.

Overall, appropriate identification and management of bilateral cryptorchid rams may contribute to improved reproductive control, animal welfare, and economic efficiency in sheep production systems.

## 5. Conclusions

The present study indicates that cryptorchidism in Sarda sheep occurs at a relatively low incidence under the conditions evaluated. Retained testes in lambs were lighter, more rounded, and morphologically distinct from those of intact animals, while adult bilateral cryptorchid rams exhibited markedly reduced testicular size as assessed by ultrasonography. No spermatozoa were detected in any recovered ejaculates, consistent with azoospermia and confirming the absence of functional spermatogenesis in these animals.

Despite their infertility, cryptorchid rams displayed preserved sexual behavior under the study conditions and appeared able to detect estrous ewes. These results suggest that, although cryptorchids cannot be used for breeding, their natural infertility and preserved sexual behavior make them potential candidates for safe, sustainable use in estrous detection. This suggests a possible zootechnical application in repurposing cryptorchid rams, a perspective rarely addressed in previous studies. However, it is recommended to test the rams for their sexual behavior and ability to detect estrous females prior to their use in reproductive activities, as well as the absence of venereal diseases. Future studies should assess endocrine profiles to better understand the basis of the preserved sexual behavior observed in bilateral cryptorchid rams.

## Figures and Tables

**Table 1:** Testis shape metrics (mean ± SD) in intact and cryptorchid lambs, with mixed-effects model *p*-values. Values represent individual testes (Intact: *n* = 20; Cryptorchid: *n* = 19; total = 39 testes from 20 animals).

Metric	Intact (*n* = 20)	Cryptorchid (*n* = 19)	*p*-Value	Hedges’ g
Aspect ratio (W/L)	0.66 ± 0.05 ^a^	0.71 ± 0.06 ^b^	0.010	−1.04
Eccentricity	0.75 ± 0.04 ^a^	0.70 ± 0.06 ^b^	0.008	1.06
Circularity	0.94 ± 0.02 ^a^	0.96 ± 0.02 ^b^	0.017	−0.99
Weight (g)	3.67 ± 0.71 ^a^	2.90 ± 0.94 ^b^	0.037	0.90

Values within rows sharing different superscript letters (a, b) differ significantly (*p* < 0.05).

**Table 2:** Testicular linear dimensions (mean ± SD) in intact and bilaterally cryptorchid rams, with *p*-values derived from linear mixed-effects models and corresponding effect sizes (Hedges’ g). Values represent per-testis observations (Intact: *n* = 7; Cryptorchid: *n* = 15). Measurements in intact rams were obtained using calipers, whereas measurements in bilateral cryptorchid rams were obtained by ultrasonography.

Metric	Intact (*n* = 7)	Cryptorchid (*n* = 15)	*p*-Value	Hedges’ g
D1 (mm)	103.50 ± 11.46 ^a^	45.45 ± 6.42 ^b^	<0.001	6.70
D2 (mm)	160.93 ± 14.69 ^a^	54.11 ± 8.71 ^b^	<0.001	9.41
D3 (mm)	73.00 ± 3.94 ^a^	30.18 ± 5.19 ^b^	<0.001	8.74

Values within rows sharing different superscript letters (a, b) differ significantly (*p* < 0.001).

**Table 3:** Testis shape metrics (mean ± SD) in intact and bilaterally cryptorchid rams, with *p*-values derived from linear mixed-effects models and corresponding effect sizes (Hedges’ g). Values represent per-testis observations (Intact: *n* = 7; Cryptorchid: *n* = 15). Measurements in intact rams were obtained using calipers, whereas measurements in bilateral cryptorchid rams were obtained by ultrasonography.

Metric	Intact (*n* = 7)	Cryptorchid (*n* = 15)	*p*-Value	Hedges’ g
Aspect ratio (W/L)	0.71 ± 0.09 ^a^	0.67 ± 0.12 ^a^	0.416	0.33
Eccentricity	0.69 ± 0.11 ^a^	0.72 ± 0.13 ^a^	0.590	−0.24
Circularity	0.95 ± 0.03 ^a^	0.93 ± 0.06 ^a^	0.180	0.46

Values within rows sharing the same superscript letter (a) are not significantly different (*p* ≥ 0.05).

## Data Availability

The data presented in this study are available on request from the corresponding authors.
